# What does antimicrobial stewardship look like where you are? Global narratives from participants in a massive open online course

**DOI:** 10.1093/jacamr/dlab186

**Published:** 2021-12-28

**Authors:** Vrinda Nampoothiri, Candice Bonaconsa, Surya Surendran, Oluchi Mbamalu, Winnie Nambatya, Peter Ahabwe Babigumira, Raheelah Ahmad, Enrique Castro-Sanchez, Alex Broom, Julia Szymczak, Walter Zingg, Mark Gilchrist, Alison Holmes, Marc Mendelson, Sanjeev Singh, Monsey McLeod, Esmita Charani

**Affiliations:** Department of Infection Control and Epidemiology, Amrita Institute of Medical Sciences, Amrita Vishwa Vidyapeetham University, Kochi, Kerala, India; Division of Infectious Diseases and HIV Medicine, Department of Medicine, Groote Schuur Hospital, University of Cape Town, Cape Town, South Africa; Department of Infection Control and Epidemiology, Amrita Institute of Medical Sciences, Amrita Vishwa Vidyapeetham University, Kochi, Kerala, India; Division of Infectious Diseases and HIV Medicine, Department of Medicine, Groote Schuur Hospital, University of Cape Town, Cape Town, South Africa; Department of Pharmacy, Makerere University, Kampala, Uganda; Infectious Diseases Institute, Makerere University, Kampala, Uganda; Division of Health Services Research and Management, School of Health Sciences, University of London, London, UK; Division of Health Services Research and Management, School of Health Sciences, University of London, London, UK; Department of Sociology and Social Policy, School of Social and Political Sciences, The University of Sydney, Sydney, Australia; Department of Biostatistics, Epidemiology and Informatics, Perelman School of Medicine, University of Pennsylvania, Philadelphia, PA, USA; Division of Infectious Diseases and Hospital Epidemiology, University Hospital Zurich, Zurich, Switzerland; Department of Pharmacy, Imperial College Healthcare NHS Trust, London, UK; Health Protection Research Unit in Healthcare Associated infections and Antimicrobial Resistance, Department of Medicine, Imperial College London, London, UK; Division of Infectious Diseases and HIV Medicine, Department of Medicine, Groote Schuur Hospital, University of Cape Town, Cape Town, South Africa; Department of Infection Control and Epidemiology, Amrita Institute of Medical Sciences, Amrita Vishwa Vidyapeetham University, Kochi, Kerala, India; Health Protection Research Unit in Healthcare Associated infections and Antimicrobial Resistance, Department of Medicine, Imperial College London, London, UK; Division of Infectious Diseases and HIV Medicine, Department of Medicine, Groote Schuur Hospital, University of Cape Town, Cape Town, South Africa; Health Protection Research Unit in Healthcare Associated infections and Antimicrobial Resistance, Department of Medicine, Imperial College London, London, UK

## Abstract

**Background:**

Whilst antimicrobial stewardship (AMS) is being implemented globally, contextual differences exist. We describe how the use of a massive open online course (MOOC) platform provided an opportunity to gather diverse narratives on AMS from around the world.

**Methods:**

A free 3 week MOOC titled ‘Tackling antimicrobial resistance: a social science approach’ was launched in November 2019. Learners were asked specific questions about their experiences of AMS via 38 optional free-text prompts dispersed throughout the modules. Content analysis was used to identify key emerging themes from the learners’ responses in the first three runs of the MOOC.

**Results:**

Between November 2019 and July 2020, 1464 learners enrolled from 114 countries. Overall, 199 individual learners provided a total of 1097 responses to the prompts. The diverse perspectives describe unique challenges present in different contexts including ill-defined roles for pharmacists and nurses in AMS; inadequate governance and policy inconsistencies in surveillance for antibiotic consumption and antimicrobial resistance (AMR) in some countries; lack of ownership of antibiotic decision-making and buy-in from different clinical specialties; and human resource and technological constraints. Patients’ knowledge, experiences and perspectives were recognized as a valuable source of information that should be incorporated in AMS initiatives to overcome cultural barriers to the judicious use of antibiotics.

**Conclusions:**

Analysis of learner comments and reflections identified a range of enablers and barriers to AMS implementation across different healthcare economies. Common challenges to AMS implementation included the role of non-physician healthcare workers, resource limitations, gaps in knowledge of AMR, and patient engagement and involvement in AMS.

## Introduction

Antimicrobial resistance (AMR) is a silent pandemic that requires urgent multisector action.^1^ The WHO-endorsed Global Action Plan on AMR provides guidance for countries to develop strategies to tackle AMR, including implementation of antimicrobial stewardship (AMS) programmes. Individual countries are at different stages of implementing national action plans across sectors, driven amongst other things by existing capacity, resource limitations and political factors.^2^^,^^3^ Effective AMS requires a multimodal and interdisciplinary approach to changing behaviours and aims to optimize antibiotic use and preserve their efficacy.^4^^,^^5^ Whilst most evidence continues to be generated from high-income countries,^6^ increasingly positive outcomes associated with AMS are being reported from low- and middle-income countries (LMICs).^7–9^

To effectively optimize antibiotic use, AMS should be implemented across primary, secondary, and tertiary sectors. Multidisciplinarity in AMS teams is important.^5^ AMS strategies include effective processes for surveillance, access to policies and guidelines, and education and training for AMS teams as well as for other healthcare workers (HCWs).^10^^,^^11^ Whilst guidelines, policies and global and national action plans exist, significant differences remain in AMS strategies, including differences in team composition and in indicators used to measure success.^7^^,^^12–14^ Whilst a multidisciplinary approach is promoted, in some countries, AMS continues to be led by doctors with little input from other healthcare professionals e.g. pharmacists and nurses, despite their potential for active roles in AMS. Furthermore, AMS initiatives rarely involve patients.^15^

Antibiotic prescribing is a complex, social process reliant on different people and influenced by determinants such as the opinions of peers and hierarchies that exist within clinical teams.^16^ In the last 10 years, a growing body of literature applying social science approaches has provided insight into the impact of behavioural and social norms on antibiotic prescribing in different contexts.^17–19^ Effective use of theories, frameworks and methods from behavioural and psychological sciences, however, remain inaccessible to most AMS practitioners.^20^ Harnessing the growing body of qualitative literature on this topic, we brought together key research expertise to develop a 3 week massive open online course (MOOC) titled ‘Tackling antimicrobial resistance: a social science approach’ (https://www.futurelearn.com/courses/social-science-for-tackling-antimicrobial-resistance) to make such approaches more accessible to AMS practitioners. This introductory course focused on the practical and real-world application of social science methods using examples of clinical practice and research from high-income countries and LMICs.

MOOCs, which enable learners to complete courses at their own pace, have gained popularity for providing affordable access to education to a wider audience.^21^^,^^22^ In this article, we describe how the use of a MOOC platform provided an opportunity to gather diverse narratives on AMS from around the world, in a large number of contexts and experiences of developing and implementing AMS. These narratives also gave us fresh insights into the unique challenges that HCWs face in implementing AMS across diverse cultural and economic settings.

## Methods

The MOOC was funded by the Economic and Social Research Council (ESRC) to enhance the impact of existing ESRC-funded research to a wider global audience, including those in LMICs. Recognizing that existing e-learning resources at the time did not address the use of social science methodologies to tackle AMR, the content was specifically developed to address this gap. The content was designed to complement the existing WHO and BSAC e-learning initiatives.^22^ Drawing on state-of-the-art evidence from application of social science research to tackling AMR across different countries, the international faculty represented expertise across social sciences, infectious diseases (ID), implementation science, pharmacy, patient and public advocacy, nursing, general practice and knowledge mobilization.

The open access course, hosted on an established platform with wide global reach, linked to existing BSAC MOOCs and targeted healthcare professionals, researchers and students. It was designed as an interactive module that uses a range of techniques such as video case presentations interspersed with knowledge tests to enhance participant engagement and learning. Each week had 2 h of materials which the learners could finish at their own pace. Week one of the course included in-depth discussions on structure, functioning and challenges faced in AMS implementation from experts across high- income countries and LMICs. Week two introduced how social science methodologies can be used to study AMR and included practical sessions by researchers from different parts of the world. Week three introduced the learners to implementation science and discussed the role played by patients and the public in AMR.

Throughout the course, learners were encouraged through prompts to share their experiences and to interact with topic-specific questions. The lead educators of the course periodically responded to comments from the learners. The course included optional free text prompts (38 in total), placed throughout the learning material and visible to all learners. The interactions between learners and educators were predominantly in response to these prompts, which, amongst others, included questions about the composition of AMS teams, the various initiatives undertaken by the AMS teams and recommendations to improve AMS activities. We extracted and analysed data in these fields to gain insights about learners’ experience and views across countries and settings. Learners’ free text responses, including their interaction with educators, from the three course runs were collated and coded in NVivo 12 using a conventional content analysis approach by four researchers.^23^^,^^24^ These codes were analysed by the researchers to identify the composition of and challenges to AMS. The purpose of this analysis is to present the information provided by the learners in their responses and not to compare the perspectives between learners and across countries. Basic learner demographic data including country, age and occupation were collected from the MOOC platform database.

### Ethics

This evaluation study was reviewed by the research office of Imperial College London who confirmed that further research ethics approval was not required.

## Results

### General characteristics of learners

Between November 2019 and July 2020, 1464 learners from over 114 countries joined the MOOC. Of the learners who provided their ages, the largest proportion (443/1464, 30.2%) were in the 26–35 years age category. There were 754/1464 (51.5%) learners from high-income countries, 646/1464 (44.1%) from LMICs and 64/1464 (4.4%) did not register their country. Out of the total learners, 199/1464 (14%) posted at least one comment on any step of the course. These included healthcare professionals such as doctors, pharmacists and nurses; students, mainly medical, nursing and pharmacy; and researchers. Learners did not consistently mention the healthcare setting or country they were from. Since the demographic data were collected anonymously it was not possible to link the individual learner comments to their demographic data. A total of 1097 comments by learners were included in the analysis.

### Overview of learners’ responses

In general, comments tended to be brief or focused on a few key points in response to the prompts, with some further clarifications amongst learners and educators as part of the discourse. Analysis of the discourse generated through the prompts identified key themes that impact AMS delivery: AMS team’s composition and activities; ill-defined roles for nurses and pharmacists; key challenges to implementing AMS strategies; and roles of the patient and the public in AMS. In the following sections, we describe the key emerging themes. Additionally, the learners, through their own experiences, had a series of recommendations through which AMS strategies could be improved. We present these at the end of the results.

### AMS teams: composition and activities

**Figure 1.**
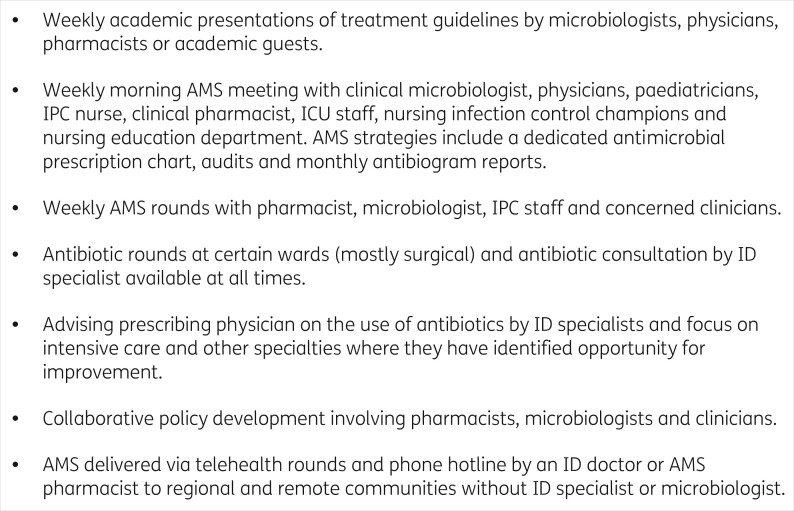
Example of AMS activities reported by learners. These report on responses by the learners and may be limited in detail to provide insight into specific contexts.

Whilst at least some components of AMS are reported to exist to varying degrees in different countries (X1, X2, X3, Table 1), some learners reported the absence of stewardship programmes in the places where they work (X4, X5, X6, Table 1). Guidelines and policies do exist (X7, X8, Table 1) though are not necessarily always put into practice (X9, X10, Table 1). A lack of guidelines (X11, X12, Table 1) was reported by some learners. The composition of AMS teams also varied (X1, X2, X8, Table 1). Learners described a range of AMS activities aimed at supporting and guiding appropriate antibiotic prescribing and use through leadership and input to clinical teams (Figure 1).

**Table 1.** Illustrative excerpts from learners’ responses on the MOOC platform about antimicrobial stewardship where they are

Theme	ID	Learner’s quote
AMS teams: composition and activities	X1	‘Weekly Friday morning AMS meeting with Clinical microbiologist, Physicians, Pediatricians, IPC Senior, Clinical Pharmacist, intensive care unit staff, nursing infection control champions and Nursing Education Department. Strategies include antimicrobial prescription chart, audits and monthly antibiogram presentation.’
	X2	‘AMS for regional and remote communities without ID/Microbiology. Delivered via tele health rounds and a phone hot line to an ID doctor or AMS pharmacist. Interventions include consulting AMS if intravenous (IV) antibiotics prescribed over 48 hours, IV to oral switch procedures, etc.’
	X3	‘Tertiary care hospitals generally have AMS but programs in long-term care or community practice lag. Provinces like Alberta have a provincial AMS. They also have the community-based ‘Do Bugs Need Drugs’ program, as does British Columbia which does some great public education and hosts an online dashboard display for AMR and antibiotic utilisation data. Ontario has a gold standard AMS scaled-up in hospitals across the province, which began in intensive care units. ‘Rx Files’ is an academic detailing program from Saskatchewan which supports stewardship decision making through consultations with physicians using the nudge method. Some jurisdictions are looking at systems for audit and feedback for prescribers.’
	X4	‘AMS in my country, Nigeria, has been underestimated in the past, only until recently that the Global Action Plan to reduce antimicrobial resistance was published and became a template which various countries around the world adopted and constitute the national version of the plan. AMS is not a term often used in this country, because, not so many even know about it.’
	X5	‘As far as I am aware, there are no stewardship activities happening in my city at any hospitals. Only IPC activities are in practice, only at large corporate hospitals who are forced to implement IPC for accreditation purposes like the Indian NABH (National Accreditation Board for Hospitals). Data collection not done expect at certain research institutes.’
	X6	‘Very little is being done or practically there are no existing structures/interventions in my environment to regulate or optimise prescription of antibiotics.’
	X7	‘We have empirical treatment and prophylaxis guidelines and an antibiotic prescribing policy which encourages the start smart then focus approach to prescribing as well as encouraging use of narrow spectrum antibiotics.’
	X8	‘Antimicrobial resistant microorganisms are increasing in our setting as people can buy antibiotics from the pharmaceutical shop without prescription. Our hospital has a yearly local antibiogram depending on the culture isolates from microbiology department. All the heads of specialties are involved in the antimicrobial stewardship committee and the antimicrobial stewardship committee develops the antibiotic prescribing guidelines based on the local antibiogram. Every year, the committee updates the antibiotics guidelines. The application of antibiotic guidelines was assessed by doing a small research of the junior doctors which was reported to the committee. We don’t have electronic prescription systems and clinical decision support systems. The committee tried to check antibiotics utilisation by global point prevalence surveys even though we don’t have a clinical pharmacist. The senior nurse is involved in the infections control committee. And continuing monitoring and education is held monthly in the hospital to improve the knowledge and current trend of antibiotics, outbreak tracing and to solve some problems. With the help of all participating departments, we can make a system to encourage the judicious use of antibiotics.’
	X9	‘At the setting that I work, there is an AMS committee. There is an antibiotic policy which is under-utilised. There are physicians who aspire to rationalise antibiotic use. Still unable to implement it due to multiple factors. I would like to see a change in attitude towards prescription of antimicrobials.’
	X10	‘Several policies and guidelines do exist, but they play little role in informing antibiotics prescription by clinicians both in rural and urban settings. The gap between policy making and implementation of guidelines should therefore be bridged by the motivated healthcare team involving the nurses and doctors and pharmacists.’
	X11	‘There are no strict measures as regard the prescription and usage of antibiotics in my country; there are only unimplemented policies. Nurses and pharmacists actively get involved in the prescription of antibiotics. I look forward to a setting where everything will be orderly. With me and other people taking this course.’
	X12	‘There are no clear policies or guidelines regarding antibiotics prescribing or purchasing and doctors recommend the antibiotics as a dose to every person for effective results and get a lot of commission by prescribing the antibiotics.’
Ill-defined roles for nurses and pharmacists	X13	‘Pharmacist have the role of monitoring of the use of antibiotics and biologist the role of monitoring of antimicrobial resistance. Data provided by pharmacist and biologist are included in a national survey about consumption of antibiotic and antimicrobial resistance.’
	X14	‘Pharmacists validate each prescription of antibiotics and advise on the indication of restricted antibiotics.’
	X15	‘Pharmacists do play a key role in AMS because they are one of the gatekeepers in terms of community’s antimicrobial access.’
	X16	‘Pharmaceutical staff often lack training and knowledge on antimicrobial drugs and AMR which exacerbates the issue of over-prescribing.’
	X17	‘Nurses and pharmacists have a limited role, which does not include the authority to make decisions regarding the person’s intake of antibiotics, but only for the nurses to administer it and for the pharmacist to provide it.’
	X18	‘Nurses and pharmacists have a role to play which is basically to educate the patient.’
	X19	‘Pharmacist are trying hard to educate the healthcare professionals and patients that misuse of antibiotics will develop resistance against bacteria.’
	X20	‘The role of nurses is not explicit. They are expected to warn of signs of infection, response to treatment, to obtain the relevant samples in a timely manner. But it has not been reflected in any document or policy. In fact, the infection control team seems to also fight against this circumstance to get the nurses involved.’
	X21	‘The nurse has a role to educate patient about the use of antibiotics, they actively remind the patient to take their medication (in the hospital).’
Challenges to implementing AMS	X22	‘People laugh at the statistics that by 2050, 10 million people will be dying every year… And I think that’s my biggest risk right now, that people still don’t take AMR as seriously as they should. The problem is not close enough to them, personally, for most people to engage with it properly. Also, there is a lack of understanding that each of us is what - 10% mammalian DNA and 90% microbial? Every creature has its own microbiome, which differs according to site. At each site it serves a defensive purpose. Disrupt it, and new problems emerge. Maybe a new perspective is required, that each of us must care for our microbial cells as well as the mammalian ones of each organ system.’
	X23	‘There is a national action plan to combat AMR, yet, the campaign is at zero level.’
	X24	‘Some countries in the region do have guidelines but it's the implementation where the problem lies. Most of these guidelines are focused on public health and not much on animal health.’
	X25	‘Several policies and guidelines do exist, but they play little role in informing antibiotics prescription by clinicians both in rural and urban settings. The gap between policy making and implementation of guidelines should therefore be bridged by the motivated healthcare team involving the nurses and doctors and pharmacists.’
	X26	‘Many of the challenges in Uganda are not more different than in other countries: lack of leadership, the lack of expertise at health centres and problems with tracking and reporting.’
	X27	‘A lot of practices described are familiar. Surgeons like to outsource antibiotic prescribing to others like internal medicine specialists, anaesthesiologists, or IDs. Once, when being consulted about a patient, the resident surgeon even said to me: we operate, but don’t know anything about the antibiotics. That’s your job to figure out which antibiotic to give, not ours.’
	X28	‘There are antibiotic stewardship rounds in surgical departments, but internal medicine etc are still not on-board with this.’
	X29	‘Healthcare associated infection data are poorly captured. Improvements are needed in communication and understanding of differences in team dynamics and AMS in different clinical areas.’
	X30	‘At present, reports from the AMS committee for our hospital is not readily available. As mentioned by the Uganda AMS scientists, we cannot work on AMR or AMS without data. I now have the buy-in from my hospital’s infection control team to work on prevention and surveillance on MRSA, and will try to get to know the AMS team of my hospital better to get the buy-in to work together.’
	X31	‘Economic analysis has not been done in my setting hence impact not realised. Also, inconsistent antibiotic ward rounds noted.’
The role of patients and the public in AMS and the wider AMR landscape	X32	‘I think, patients view or experiences are important in carrying on with a successful intervention or modifying it.
		Patients perspective can add to the knowledge of prescribing or a treatment plan as the one going through the experience is the patient and not the healthcare professional.’
	X33	‘End-users of antimicrobial drugs are a fundamental part of the whole AMS process so yes, their input must be included wherever possible, as part of understanding the context in which interventions are to operate. I think that this would alter the perspective and focus of some decisions. Policies and guidelines are all very well but they have to have the desired effect, so it seems relevant to assess key performance indicators and then work backwards using social science methods to identify which changes can be made which could have the biggest positive effect.’
	X34	‘The answer for using more patient’s knowledge and experience in my daily practise is yes, I would. Beside good medical records of each patients, it’s also needed to explore more about how much they understand their issue/case so we can fill the gap of the missing essential information for them.’
	X35	‘Patient involved would be vital for designing and reviewing interventions and materials that are directly targeted at patients and citizens. Patient involvement in wider interventions could also throw up useful questions that health professionals haven't considered (e.g. communication, risks, etc). This information could be gathered at intervention design meetings, through consultation, and direct discussions with expert patients.’
	X36	‘My local GP practice has been very ahead of its time when it comes to AMR and has put in place strategies to avoid over prescription of them. I have heard of stories in the past of patients requesting antibiotics and sometimes even requesting them ‘just in case’ they’re infection became worsened, sometimes people would request them to take abroad if they were prone to some infection. Therefore, in the waiting room there are posters placed to educate people of the potential risks to unnecessary prescription of antibiotics. The people in my community have started to understand the issue with AMR. The GPs now avoid their prescription unless in dire need.’

### Ill-defined roles for nurses and pharmacists

A recurring response from learners is that the distinct role of nurses and pharmacists in AMS remains ill defined. A summary of challenges in AMS across sectors, as it relates to pharmacists’ and nurses’ roles, is presented in Table 2. Pharmacists offer both patient-by-patient ground level view and hospital-wide perspective on antibiotic use and consumption (X13, Table 1). Acting as gatekeepers, pharmacists review and authorize antibiotic prescriptions and provide advice on the indication of restricted antibiotics in hospital (X14, X15, Table 1). Some learners ascribe the lack of training and knowledge on antimicrobial drugs and AMR and the restricted/limited perception of the pharmacist’s role by colleagues as barriers in their active role and contribution to AMS (X16, X17, Table 1). As medicines expert, pharmacists are ideal candidates to provide education to healthcare professionals and patients on antimicrobial use (X18, X19, Table 1).

**Table 2.** Reported roles and challenges of pharmacists and nurses in AMS across sectors^a^

	Pharmacists	Nurses
Roles (setting included when known)	Review and validate each prescription of antibiotics. Advise on and authorize the appropriate use of restricted antibiotics. Educate and advise HCWs and patients about the rational use of antibiotics and AMS. Monitor antibiotic use and provide data on antibiotic consumption. Collect data on AMS performance indicators and provide feedback to stakeholders. Act as gatekeepers for antimicrobial access in the community. Facilitate communication between a doctor and a patient.	Administer antibiotics. Prescribe antibiotics in contexts where it is permitted. Monitor patients and respond to signs of infection. Educate patients about the use of antibiotics and ensure that prescribed courses are completed by patients in hospital.
Challenges	Lack of training and knowledge on antimicrobials and AMR, which exacerbates the issue of over-prescribing. Perception that pharmacists have a limited role in antibiotic decision-making as their role is restricted to dispensing antibiotics. Providing antibiotics without a prescription to patients who are unable to see a doctor.	Perception that nurse’s role in AMS is limited to antibiotic administration. Despite nursing unit managers and ward champions attending AMS rounds, their roles are not defined. Apart from IPC nurses, the general nursing body is not represented in AMS committees. The role of nursing in AMS is not explicit. They are expected to warn of signs of infection and response to treatment, and to obtain the relevant samples in a timely manner. These have not been reflected in any document or policy.

a These report on responses by the learners and may be limited in detail to provide insight into specific contexts.

The role of nurses in AMS is not clearly defined in policies or guidelines. Even though the nurse’s role in AMS is perceived to be limited to antibiotic administration (X17, Table 1), routine nursing roles also include monitoring and reporting response to antimicrobial treatment and early signs of infection as well as obtaining relevant samples in a timely manner (X20, Table 1). Learners report that nurses play an active role educating patients about the use of antimicrobials and ensure that prescribed courses are completed while they are in hospital (X18, X21, Table 1).

### Challenges to implementing AMS across settings

**Figure 2.**
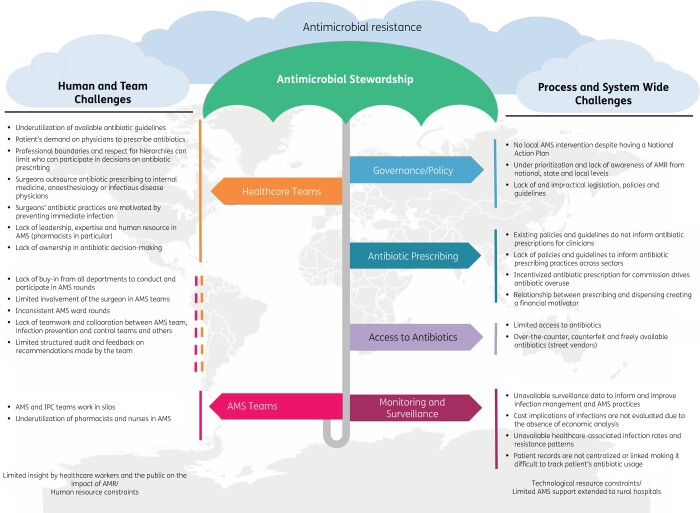
Reported challenges and limitations to AMS.

Figure 2 depicts a summary of responses from learners on the perceived challenges of implementing AMS across different contexts and settings, grouped into team- and systems-based challenges. In general, learners perceived that the public and HCWs have limited awareness on the impact of AMR on human health and did not perceive it as an actionable problem that they could play a role in (X22, Table 1). The impact of AMR is under-prioritized in some settings where learners describe a lack of emphasis, awareness and priority from national, state and local authorities. Discrepancies exist between national action plans and actual interventions to combat AMR (X23, X24, X25, Table 1).

A few learners reported unavailability of AMS-specific policies and protocols, while some others stated that where they do exist, they do not always target antibiotic prescribing across both primary and secondary care (X25, Table 1). AMS support to rural hospitals was described as limited. The shortage of clinical pharmacists and the lack of leadership and expertise on AMS highlights human resource and capacity challenges while technological constraints include the absence of electronic prescription systems (X8, X26 Table 1).

Whilst unregulated access to antibiotics is a recognized concern, a myriad of other factors impacts on their optimized use even when they are regulated. Some learners observe a lack of ownership of antibiotic stewardship practices by prescribers and members of their healthcare teams and list, among others, several behavioural approaches/challenges to antibiotic prescribing that affect decision-making (X27, Table 1). The gap in the clarity of roles and expectations by other HCWs of AMS teams, together with how the interface across other HCW and AMS teams is described, points towards challenges to AMS that include varied prescribing practices and lack of buy-in (X28, Table 1).

Inconsistent surveillance strategies are reported by learners. Surveillance data on antibiotic consumption, healthcare-associated infections and resistance patterns to inform or improve infection management practices are infrequent, poorly captured or absent (X29, X30, Table 1). These inconsistencies are further challenged by an absence of or limited and/or inadequate audit and feedback loops to improve processes. While there are many serious short- and long-term consequences on patient outcomes and AMR resulting from suboptimal surveillance, learners also highlight the hidden financial implications of infections due to the absence of economic data and analysis (X31, Table 1).

### The role of patients and the public in AMS and the wider AMR landscape

Learners acknowledged that patients have a key role to play in AMS as they are the consumers of antibiotics and beneficiaries of health services (X32, Table 1). As the main source of continuity, a patient’s perspective can provide invaluable insight into past treatment plans and contribute knowledge that can potentially enhance the success of future treatment options identified by the clinician (X33, Table 1). There is also a need to explore how much patients understand their own care needs so that health communication can be tailored to their needs (X34, Table 1). In addition to involving patients in their care, patient involvement in the wider development and evaluation of interventions is essential as it will highlight needs that healthcare professionals haven't considered (e.g. communication, risks) (X35, Table 1). With unregulated access to antibiotics a concern in many countries, learners suggest that education on the adverse effects of unnecessary antibiotic use should be provided to patients to tackle AMR (X36, Table 1).

### Recommendations for improvement

**Figure 3.**
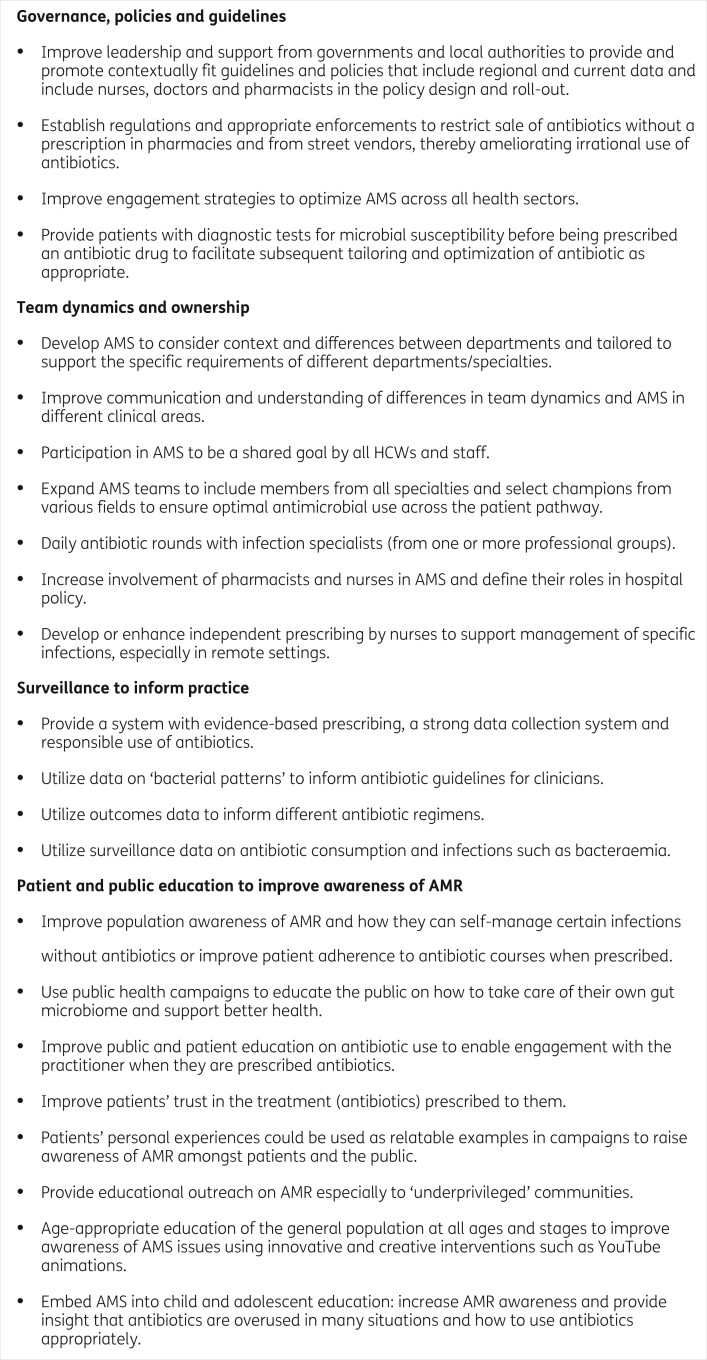
Learners’ recommendations for improvements in AMS programmes based on their own experiences.

Learners also put forth various recommendations for how AMS can be optimized and what elements need to be included. Figure 3 summarizes these recommendations. This is a valuable contribution as it is a reflection of the perspectives of diverse healthcare professionals on how AMS can be better designed and implemented. Furthermore, these recommendations highlight the existing disparities on the shape and role of AMS in different healthcare settings.

## Discussion

In this article, we analysed responses from learners around the world who enrolled in a 3 week long MOOC on applying social science methods to tackle AMR. The responses yielded rich data on the unique challenges and experiences of developing and implementing AMS across different countries. The reported challenges to implementing AMS strategies include: limited awareness on the impact of AMR on human health amongst the HCW and general populations; lack of governance and policy; insufficient surveillance for antibiotic consumption and AMR; human resource and technological constraints; variable access to essential antibiotics; and lack of ownership of antibiotic decision-making and buy-in from different clinical specialties. Patients’ knowledge, experiences and perspectives were recognized as valuable in the consideration of AMS initiatives.^25^

Even though AMS is a universally accepted strategy for rationalizing antibiotic use, our findings suggest there is still a critical lack (or perhaps visibility) of policies and guidelines in some places. Even when these are in place, there are still limitations in the way AMS is implemented in different settings. Delivering AMS through a multidisciplinary team was viewed to be desirable; however, it may not be feasible in all contexts especially due to resource constraints. Pharmacist-driven or pharmacist-led AMS programmes have been shown to improve antimicrobial prescriptions where there is a lack of availability of ID specialists.^26^^,^^27^ However while it was recognized by the learners that involving pharmacists and nurses in AMS is beneficial, they believed the distinct roles remain ill-defined in many countries, creating a potential obstacle in the implementation of global AMR priorities. Even though theoretical advances from academia or policy describe nurses’ roles, these are yet to be translated to clinical settings.^28^^,^^29^ Published evidence also suggest that the extent to which these professionals are involved remains a barrier; ^30^^,^^31^ however, we recognize not all pharmacists and nurses may be suitably trained for AMS activities, and, where possible, should involve those with specialist knowledge/skills. Patient education on rational antibiotic use was an area that the learners acknowledged pharmacist and nurses to have an important role.

In general, learners report that the public and HCWs have limited awareness of the impact of AMR on human health. This may be due to many reasons and may be linked to the lack of government leadership and efforts from healthcare organizations to improve this awareness. In some learners’ countries, the impact and effect of AMR were perceived as not being prioritized and learners describe a lack of emphasis, awareness and significance attributed to this from national, state and local authorities. A qualitative study investigating cultural and contextual determinants of AMS across different countries found that government or state involvement could be both a help or hindrance to effective AMS, e.g. in high-income countries, too much interference caused conflicting messages and disruption to AMS,^32^ leading to uncoordinated and unfocused messages risking ‘AMR fatigue’. Conversely, in LMICs, the lack of government support and poor infrastructure were considered barriers to AMS. An interesting outcome was that irrespective of income status or central governance endorsement, local championing and leadership was considered a key facilitator to successful AMS implementation.^32^

Difficulty in implementing AMS in rural areas has been reported in other studies.^33^^,^^34^ In a mixed-methods study to identify barriers and enablers for implementing AMS in regional and rural hospitals in Australia, barriers include lack of access to education, resources and specialist support.^33^ To reiterate the influence of resource availability on AMS in rural district facilities, a situational analysis reviewing existing AMS facilities in a South African province reported that AMS was less likely to be established in rural districts with smaller facilities and smaller staff complements.^34^ Specialist onsite support, which includes ID, clinical microbiology and pharmacy,^33^^,^^35^^,^^36^ is deemed key to success for the development of AMS programmes but may not be feasible or possible in many settings, thus alternative models of work should also be explored. Where feasible, outcomes from pharmacist-led interventions conducted in several countries in Africa have demonstrated improvements including better hang-time compliance and a reduction in surgical site infections and antibiotic use, demonstrating that AMS can be implemented with limited specialist resources and extended to remote areas.^27^^,^^37^^,^^38^ Whilst a multidisciplinary AMS team remains the gold standard, existing evidence suggests having the right person lead the AMS programme may be sufficient to making a measurable difference.

The influence of the healthcare system, availability of antibiotics and diagnostic capability, and infection prevention and control (IPC) practices on AMS interventions is well described, where the discrepancies between income status are often highlighted.^6^^,^^8^^,^^9^ However, when considering antibiotic decision-making, associated behaviours linked with prescribing practices seem universal and less linked to the country’s income status.^16–18^^,^^39^ Rather, prescribing practices are influenced by cultural and contextual boundaries and practices.^4^

To facilitate effective implementation, contextualized strategies are needed.^9^ The range of reported barriers to AMS include: diagnostic challenges; varied knowledge and awareness on optimal antimicrobial use; access to antimicrobials; healthcare facilities varying in infrastructure and patient numbers; inadequacy of information systems; lack of key personnel and funding; and the competing healthcare needs of populations that drive prioritization of initiatives.^9^^,^^40^ Learners also emphasize gaps in engagement between AMS teams and the healthcare teams they consult. Published literature confirms that AMS teams can work in isolation often with limited engagement with other specialties;^4^^,^^41^ roles and expectations in AMS from the wider multidisciplinary team are unclear;^17^^,^^18^^,^^39^ and lack of buy-in with respect to AMS may be exhibited by clinicians from other departments.^39^

Poor surveillance was another challenge reported by learners as a gap to effective AMS. The benefits of surveillance are well described, and the literature reports an overall reduction in mean antibiotic use when prospective audit is applied in combination with intervention and feedback.^27^^,^^42^ Essentially, surveillance is a means to audit behaviour change. Effective, relevant and timely feedback of behaviours to measure change are recommended by Singh *et al.*^18^ as one of the components on a framework to improve integrated care in infection management.

Patients’ and the public’s role in and contribution to AMS was recognized by the learners. Value is placed on how the patient’s perspective can usefully calibrate and widen HCWs’ views on AMS to improve outcomes. A gap exists both in the active engagement of patients in decision-making to ameliorate demands for unregulated antibiotics and in greater awareness of their own infection care. Although much is written about engaging patients on IPC and AMS in policies and guidelines, a recent scoping review suggests that current infection-related patient participation measures are limited, emphasizing the many missed opportunities for patient engagement.^43^^,^^44^

This study has limitations. Whilst this MOOC enabled gathering of insights about AMS from participants across the world, responses could not be linked to the learner’s specific country as this information was not consistently available for all learners. It is likely the views expressed are biased or skewed based on personal narratives and experiences. The findings represent the experiences of individual learners participating in a MOOC and may not be generalizable to the wider context of the countries of the participants. Furthermore, learners’ comments varied in length and detail, which limited in-depth analysis. Despite these limitations, the learners’ experiences provide useful insights into AMS from diverse cultural and economic contexts.

### Conclusions

This analysis of perspectives and experiences of learners in different countries provided insights into the unique challenges present in different contexts, spanning teams and systems considerations. There need to be greater efforts in recognizing the clinical and leadership role of non-physician healthcare professionals in AMS as well as seeking greater active patient and public involvement. Customizing AMS programmes to account for contextual drivers such as local leadership structures and access to antibiotics can facilitate the adoption of sustainable interventions.
